# A unique origin of the brachioradialis muscle from the triceps brachii

**DOI:** 10.1007/s12565-025-00833-6

**Published:** 2025-03-25

**Authors:** George Triantafyllou, Fabrice Duparc, George Tsakotos, Maria Piagkou

**Affiliations:** 1 Faculty of Health Sciences, Department of Anatomy, School of Medicine, National and Kapodistrian University of Athens, 75 Mikras Asias Str., Goudi, 11527 Athens, Greece; 2 Faculty of Medicine-Pharmacy, Department of Anatomy, University of Rouen-Normandy, Rouen, France

**Keywords:** Brachioradialis muscle, Variation, Radial nerve, Anatomy, Dissection

## Abstract

Presently, a few brachioradialis muscle (BR) variants have been reported, mainly concerning their inserting tendons or the accessory forms (accessory BR muscles). During a routine dissection of a 74-year-old female donated cadaver, a unique muscle variant was observed unilaterally. An aberrant origin of the BR from the lateral head of the triceps brachii was identified on the left arm. The radial nerve (RN) superficial branch coursed posteriorly to the BR before obtaining its superficial position. On the right arm, the BR was typical. So far, some of the BR variants in its origins have been reported, such as arising from the muscular belly of the brachialis or the insertion of the deltoid muscle. Thus, the present variant could be a worth noting rare case. Furthermore, the posterior position of the RN superficial branch could have potential clinical significance and may cause entrapment neuropathy.

## Introduction

The musculoskeletal system exhibits both typical and variant anatomy. Significant research has been conducted on muscle variants in the arm due to their clinical and surgical relevance (Zielinska et al. 2022; Szewczyk et al. 2022; Piagkou et al. 2023, 2024; Herma et al. 2023; Triantafyllou et al. 2024a).

The brachioradialis muscle (BR) is the most superficial forearm muscle on the radial side. It originates from the proximal two-thirds of the lateral supracondylar humeral ridge and inserts into the distal end of the radius, proximal to the styloid process (Standring et al. 2016). The radial nerve (RN) courses between the brachialis muscle (anteriorly) and BR (posteriorly), where it can also be palpated (Standring et al. 2016). This is an important relationship due to the possibility of RN muscular entrapment (Węgiel et al. 2023). *Gray’s Anatomy* describes a few variants of the BR, such as its proximal fusion with the brachialis and the BR tendon bifurcation or trifurcation (Standring et al. 2016). *Bergman’s Comprehensive Encyclopedia of Anatomic Variations* also presents only a few variants of the BR for its origin, insertion, and accessory form (Tubbs et al. 2016).

Although it is rarely reported that one muscle originates from another muscle, the ulnar head of the pronator teres was found arising from the third head of the biceps brachii (Olewnik et al. 2023). The current report described a unique BR origin, identified during a routine dissection, with further discussions on its morphological variability and potential clinical significance.

## Case report

Dissection was performed at the Anatomy Department of the Medical School of National and Kapodistrian University of Athens on a 74-year-old female donated cadaver for educational and research purposes. The body was derived from the “Body Donation Program” (Brenner et al. 2024), and the authors confirm that every effort was made to comply with all local and international ethical guidelines and laws concerning the use of human cadaveric donors in anatomical research.

**Fig. 1:**
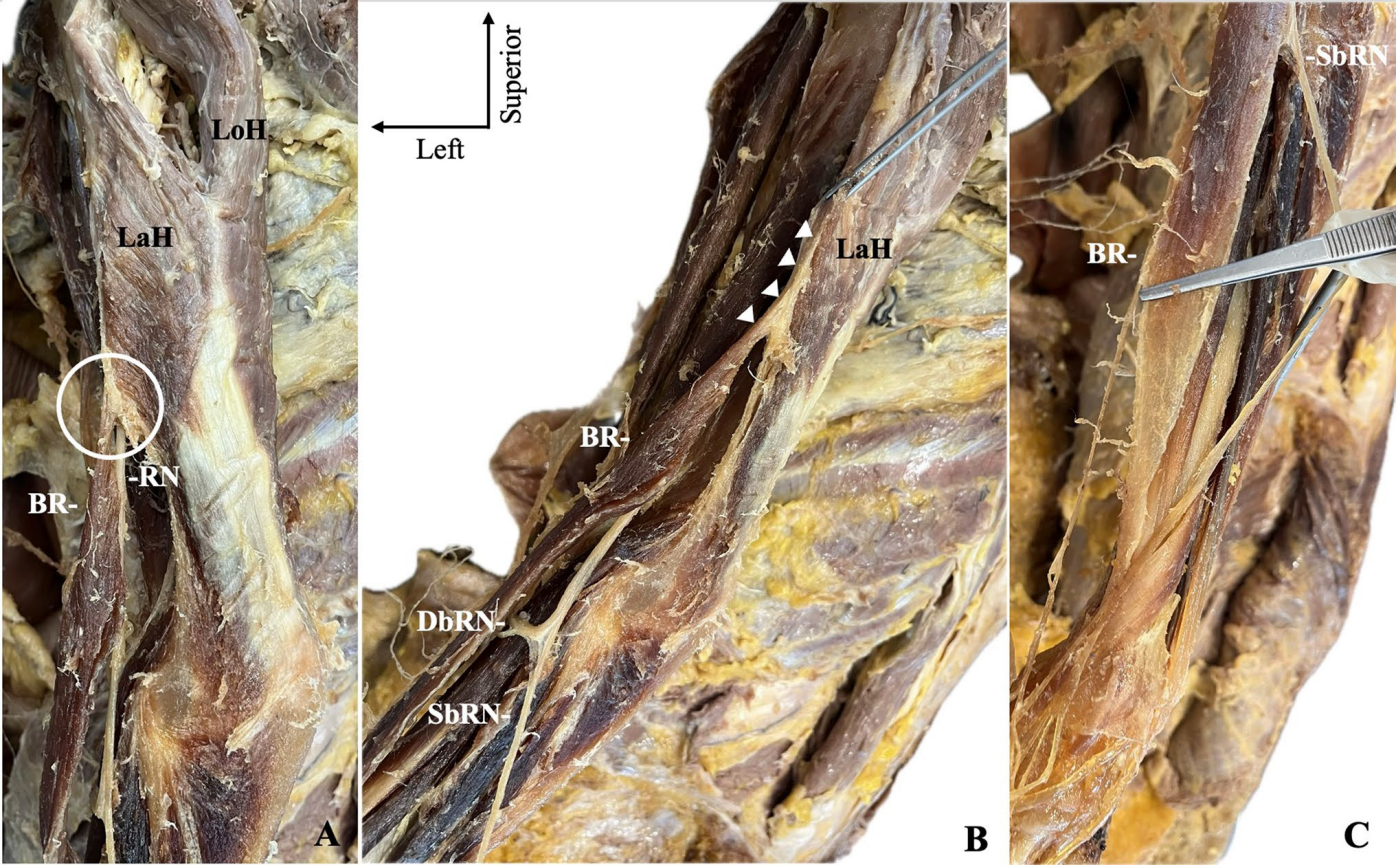
The origin of the brachioradialis muscle (BR) from the lateral head of the triceps brachii (LaH) along with the radial nerve (RN) is depicted with a circle (**A**) and arrowheads (**B**), respectively. The BR typical insertion is depicted in (**C**). *SbRN* superficial branch of radial nerve, *DbRN* deep branch of radial nerve

Skin and subcutaneous fat from the arm and forearm were excised to expose the muscles and neurovascular structures. The BR, brachialis, triceps brachii (TB) muscles, and RN were identified on the lateral compartment of the arm. On the left arm, the brachialis was identified as typical; however, the BR origin was aberrant, as determined from the TB lateral head (Fig. 1a, b). The BR was typically inserted into the styloid process of the radius (Fig. 1c). The RN was identified as piercing the lateral intermuscular septum and coursing between the TB–brachialis (before the origin of BR). At the cubital fossa, the RN divided into the deep and superficial branches, while the superficial branch coursed between the BR and the extensor carpi ulnaris. Then it obtained its superficial course (laterally to the BR) at the proximal one-third of the BR muscular belly. A limitation of this case was that the BR arterial supply was not identified. On the right arm, the BR was typical.

## Discussion

During the seventh and eighth developmental weeks, the BR becomes entirely distinguishable (Wilde et al. 2020). Developmentally, the muscle belongs to the forearm extensor muscles that differentiate from the common pre-muscular mass on the laterocephalic side of the forearm (Bardeen 1905). Three muscle groups can be observed: the superficial, radial, and deep groups (Bardeen 1905). The BR belongs to the radial muscle group, which also gives rise to the extensor carpi radialis muscle. The BR appears fused with the distal end of the radius (distally) and with the lateral epicondyle of the humerus (proximally) (Bardeen 1905). Alterations to these complex procedures could lead to muscle variants.

In the present cadaveric report, we identified an aberrant origin of the BR from the TB lateral head. In *Bergman’s Comprehensive Encyclopedia of Anatomic Variations*, the typical origin is described from the upper two-thirds of the humerus between the brachialis and TB (Tubbs et al. 2016). The origin can often be united with the brachialis, or it can extend proximally to the deltoid muscle (Tubbs et al. 2016). Macalister reported only a few variants of the muscle, mainly for its tendon, while he described an origin joined brachialis and continuous with the deltoid insertion (Macalister 1875). Testut (Testut 1884) and LeDouble (Double 1897) did not report other variants of the BR origin. Thus, we could not identify a fusion or origin of the BR from the lateral head of the TB in the literature. Nevertheless, a unique insertion of the BR was reported into the third metacarpal bone (Sañudo et al. 1996).

Although the BR origin does not present itself with a high rate of morphological variability, variants in the inserting tendon and/or accessory form are frequently reported (Tubbs et al. 2016). Rodriguez-Niedenfuhr et al. systematically investigated the accessory BR in 176 upper limbs (Rodríguez‐Niedenführ et al. 2001). They observed the accessory form in 2.8% (5 cases) with an origin from the lateral supracondylar ridge and an insertion into the radial tuberosity or the radius anterior surface (Rodríguez‐Niedenführ et al. 2001). Similar findings were recently reported, where this variant was identified in 2.4% (2 cases out of 83 upper limbs) (Triantafyllou et al. 2024a). This variant was defined as the “*brachioradialis accessorius or brevis*” due to the shorter form compared to the typical BR. Triantafyllou et al. identified a unique case of accessory BR that originated between the deltoid and TB and was inserted into the suprastyloid crest of the radius (Triantafyllou et al. 2024b). They defined this variant as the “*brachioradialis longus*” and clarified that it should be differentiated from the “*brachioradialis accessorius or brevis*” (Rodríguez‐Niedenführ et al. 2001; Triantafyllou et al. 2024a). Moreover, Herma et al. identified two cases (0.96%) with two bellies of the BR that were penetrated by an accessory superficial branch of the RN (Herma et al. 2023).

The RN can be entrapped by the BR due to their close relationship (Węgiel et al. 2023). Muscle variants can alter this relationship and expose the RN to a more prone position. When the accessory form of the muscle is present, the RN most commonly courses posteriorly to the accessory structure (Rodríguez‐Niedenführ et al. 2001; Triantafyllou et al. 2024a). Nevertheless, Piagkou et al. (Piagkou et al. 2024) described the bilateral presence of a four-headed brachialis muscle, with the RN coursing through a muscular tunnel between the accessory heads and the BR. Another interesting variant course was described by Khadanovich et al. (Khadanovich et al. 2024), where the RN superficial branch coursed through the supinator canal. When compression neuropathy affects the superficial branch, pain or numbness can be reported on the wrist and/or thumb due to the sensory distribution of the nerve (Węgiel et al. 2023; Turkof et al. 1995; Spinner and Spinner 1996).

## Conclusion

This case presents a unique BR origin from the lateral head of the TB. In this situation, the superficial branch of the RN passed posterior to the aberrant muscle before attaining its final superficial position. Because most of the variants in BR origin are from the brachialis or deltoid muscle, it is relatively rare that the BR originated from the muscle of the TB lateral head. The close anatomical relationship between this muscle and the RN carries important clinical implications, as it may lead to entrapment neuropathies. Clinicians should know these muscle variants and include them in their differential diagnoses.

## Data Availability

All the data are available upon reasonable request to the corresponding author (George Triantafyllougeorgerose406@ gmail.com).
